# Editorial: Image processing methods in animal MRI and their application to evaluate brain function

**DOI:** 10.3389/fnins.2023.1147057

**Published:** 2023-02-06

**Authors:** Shengxiang Liang, Georgios A. Keliris, Jie Wang, Baoci Shan

**Affiliations:** ^1^National-Local Joint Engineering Research Center of Rehabilitation Medicine Technology, Fujian University of Traditional Chinese Medicine, Fuzhou, China; ^2^Rehabilitation Industry Institute, Fujian University of Traditional Chinese Medicine, Fuzhou, China; ^3^Traditional Chinese Medicine Rehabilitation Research Center of State Administration of Traditional Chinese Medicine, Fujian University of Traditional Chinese Medicine, Fuzhou, China; ^4^Bio-Imaging Lab, Department of Biomedical Sciences, University of Antwerp, Antwerp, Belgium; ^5^Foundation for Research and Technology – Hellas, Heraklion, Greece; ^6^Institute of Neuroscience and Brain Diseases, Xiangyang Central Hospital, Affiliated Hospital of Hubei University of Arts and Science, Xiangyang, Hubei, China; ^7^Academy of Integrative Medicine, College of Integrative Medicine, Fujian University of Traditional Chinese Medicine, Fuzhou, China; ^8^Key Laboratory of Magnetic Resonance in Biological Systems, State Key Laboratory of Magnetic Resonance and Atomic and Molecular Physics, National Center for Magnetic Resonance in Wuhan, Wuhan Institute of Physics and Mathematics, Innovation Academy for Precision Measurement Science and Technology, Chinese Academy of Sciences, Wuhan National Laboratory for Optoelectronics, Wuhan, China; ^9^University of Chinese Academy of Sciences, Beijing, China; ^10^Beijing Engineering Research Center of Radiographic Techniques and Equipment, Institute of High Energy Physics, Chinese Academy of Sciences, Beijing, China

**Keywords:** magnetic resonance imaging, analysis method, neurological diseases, brain function, functional evaluation, animal

As a non-invasive and non-radiative technique, magnetic resonance imaging (MRI) has been widely used in brain research (Ni, 2021). Recently, more and more findings about brain activation mode in specific state were reported *via* animal models and MRI (Benveniste and Blackband, 2002). The purpose of this special topic is to provide knowledge and information on the state-of-the-art methods currently used for image acquisition, (pre)processing, and evaluation of brain function in small animals.

It is difficult to expound brain function at cellular, systematic and functional levels. Multimodal neuroimaging approaches are able to combine techniques such as calcium imaging, optogenetics, electrophysiology, chemogenetics with functional magnetic resonance imaging (fMRI) and help scientists uncover the relationship between neuronal activity and vascular network dynamics, which would promote our knowledge of the brain from the level of single cells or circuits to the whole brain (Kosten et al.). Huang et al. reviewed fMRI papers of small animals under the state of stimulation categorized into electrical, visual, olfactory, auditory and other ways, and analyzed the similarities and differences, advantages and disadvantages of the stimulation sites, electrodes, and stimulation methods. The study provided invaluable comparative information on brain fMRI of small animals combined with stimulation (Huang et al.).

Lopez-Castro et al. proposed a method for operation of long-term implantable monopolar carbon electrodes in rats. Prelimbic cortex (PRL) implantable monopolar carbon electrodes were used to treat alcohol use disorder. This method of implantation generates smaller MRI artifact and serves clinical and basic research (Lopez-Castro et al.). In terms of data processing, Bao et al. constructed an automatic method for individual parcellation of rat brain MEMRI images, which further benefited case-control statistical analysis and improved the accuracy of ROI-based imaging analysis.

Alzheimer's disease (AD) is the most common cause of dementia. Many clinical and basic studies have aimed at improving effective identification strategies for AD (Valenzuela et al., 2020). Some AD imaging articles are included in this edition. High resolution diffusion tensor imaging (DTI) scanning displayed that the fractional anisotropy (FA) of 5xFAD mice showed differences at 4 months, indicating that the FA index of DTI can be used as a sensitive biomarker to detect amyloid deposition in 5xFAD mice, and can be used for preclinical research and detection of the efficiency of AD drugs (Maharjan et al.). At the same time, resting state fMRI of AD mice after electroacupuncture(EA) at Baihui and Shenting indicated that the ReHo value of local neuronal integration in hippocampus, entorhinal cortex and other brain regions increased, as well as the number of functional connectivity and neural fiber connections between entorhinal cortex and hippocampus increased, suggesting that EA intervention may be an effective strategy to treat memory defects in AD recognition (Lin et al.).

Hippocampus is closely associated with cognition and emotion. Sepsis associated encephalopathy (SAE) has been identified as a common complication of sepsis. SAE-induced pathological changes in the hippocampus may lay the foundation for the development of cognitive dysfunction and affective disorders. Yao et al. found significant changes of hippocampal-related brain networks i-n SAE rats, and the functional connectivity between the hippocampus and thalamus was positively correlated with affective deficits. Li et al. found that increased ALFF value, Glx/Cr ratio and mI/Cr ratio in hippocampus of SAE rats were positively correlated with cognitive impairment. Thus, changes in hippocampal network, ALFF, and metabolites may be potential neuroimaging biomarkers of cognitive impairment in SAE patients, which might provide the basis for the diagnosis of clinical sepsis (Li et al.).

Pan et al. used ^18^F-FDG PET/CT to identify the subregions of the posterior splenic cortex, the role of RSCDS and RSCGS in the coding process of rat contextual fear conditioning model, and the underlying brain network mechanism. The results showed that the role of rat RSCS in the formation of scene fear memory mainly depends on the RSCDS rather than the RSCGS.

In segmental sampling of rat's sciatic nerve imaging in DTI, RS-5 EPI sequence-derived FA and RD may be highly sensitive quantitative biomarkers for detecting histopathological changes of sciatic nerve in rats. These findings provide some reference value for the optimization of DTI sequence in the future peripheral nerve MRI research (Chen et al.).

We hope that the aforementioned studies which were included in this special topic can inspire new studies and novel future applications of small animal MRI in the fields of data acquisition, analysis methods, pre-clinical applications, biomarker detection and can contribute in more accurate prediction of disease progression and bring us a step close to clinical applications.

## Author contributions

All authors listed have made a substantial, direct, and intellectual contribution to the work and approved it for publication.
